# Belief in the miracles of Lourdes: A voxel‐based morphometry study

**DOI:** 10.1002/brb3.1481

**Published:** 2019-12-20

**Authors:** Anne Schienle, Carina Höfler, Albert Wabnegger

**Affiliations:** ^1^ BioTechMed Clinical Psychology University of Graz Graz Austria

**Keywords:** miracles of Lourdes, religious–spiritual well‐being, specific religious beliefs, voxel‐based morphometry

## Abstract

**Background:**

It has been shown that particular religious beliefs and practices are associated with brain function and structure. The present voxel‐based morphometry study investigated the correlation between the belief in the miracles of Lourdes (a major Catholic pilgrimage site) and gray matter volume in specific brain regions associated with theory of mind (ToM).

**Method:**

Structural brain data from 84 women (mean age: 25 years; no current somatic illness; 80% Roman‐Catholic) were correlated with self‐report measures on belief in miracles, religious–spiritual well‐being, and psychological problems. Selected brain regions of the ToM network included the temporoparietal junction (TPJ), hippocampus, amygdala, and medial prefrontal cortex (MPFC).

**Results:**

The belief in the miracles of Lourdes was positively correlated with general religiousness and with feelings of connectedness; there was no association with psychological problems. Belief in miracles of Lourdes correlated positively with TPJ volume and negatively with MPFC volume.

**Conclusion:**

Belief in the miracles was associated with brain volume in regions involved in mentalizing and self‐control.

## Graphical abstract

The present voxel‐based morphometry study identified correlations between the belief in the miracles of Lourdes (a major Catholic pilgrimage site) and grey matter volume in specific brain regions associated with theory of mind (ToM). Relevant brain regions included the temporoparietal junction (TPJ) and the medial prefrontal cortex (MPFC).

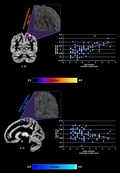

## 1 INTRODUCTION

Lourdes is a site in France that has been associated with medical miracles for the past 150 years. More than 7,000 cases of cured diseases have been recorded by the Medical Bureau of Lourdes (https://www.lourdes-france.org/en/miraculous-healings/; retrieved: September 9, 2019). Seventy of these healings (e.g., patients with previous diagnoses of tuberculosis, cancer, paralysis) have been recognized as miraculous by the Catholic Church. The most recent miracle was officially declared in 2018 (https://www.lourdes-france.org/en/miraculous-healings/; retrieved: September 9, 2019).

Many healings have been attributed to the application of water originating from the grotto in Massabielle close to the city of Lourdes. The location of the spring had been described to Bernadette Soubirous, who is also known as Saint Bernadette, in 1858. According to her testimony, she had several religious visions, during which the Virgin Mary told her to go to the spring, drink and wash and to build a chapel on the site (today known as the sanctuary of Lourdes).

Lourdes is visited year after year by thousands of pilgrims because of the reported miraculous healings reflecting the widespread belief in miracles (Harris, 1999). A survey on miracles by the Pew Research Center (a nonpartisan fact tank, USA) revealed that 79% of respondents either “completely” or “mostly” agreed that miracles occur today (Pew Forum on Religion and Public Life, 2010).

The current structural magnetic resonance imaging (MRI) study investigated neuroanatomical correlates of this type of belief in miracles. At present, only a small number of studies exist on the association between brain structure and religious beliefs or activities. The majority of these have focused on how religiously inspired techniques of meditation are associated with gray matter volume (GMV) (for a review see Tang, Hölzel, & Posner, 2015). These studies compared experienced meditators and controls or investigated changes in brain volume related to mindfulness practice. The findings pointed to meditation‐related increased volume in the hippocampus and temporoparietal cortex and reduced volume in the amygdala. The mentioned regions are involved in learning and memory (hippocampus), self‐referential processing and perspective taking (temporoparietal cortex) and decoding of emotional salience (amygdala).

A few MRI studies have focused on representations of religious beliefs in the brain (belief in God). For example, Kapogiannis, Barbey, Su, Krueger, and Grafman (2009) conducted an MRI study with healthy participants who reported different degrees and patterns of religiosity. The volume of the middle temporal cortex was positively associated with experiencing an intimate relationship with God, while GMV in the precuneus and orbitofrontal cortex was negatively associated with fear of God. In a functional MRI experiment of the same group (Kapogiannis, Barbey, Su, Zamboni, et al., 2009), the participants indicated whether they agreed or not to specific statements (e.g., “God protects all people”). Statements addressing God's involvement modulated activity in different brain regions including the inferior/medial frontal gyrus, the middle/inferior temporal gyrus, and the precuneus.

Moreover, there is evidence that brain damage and specific neurological disorders can bring about religious or spiritual experiences. Urgesi, Aglioti, Skrap, and Fabbro (2010) found that lesions of specific brain regions modulated spiritual feeling and thinking. The authors investigated patients before and after removal of their brain tumor and found that depending on the location of the removed tissue, self‐transcendence (the enduring tendency to transcend contingent sensorimotor representations and to identify the self as an integral part of the universe as a whole) was specifically altered. Surgery of parietal regions induced a significant increase in self‐transcendence. Changes in religious beliefs and behaviors have also been reported from patients with frontotemporal dementia and temporal‐lobe epilepsy (e.g., Arzy & Schurr, 2016; Devinsky & Lai, 2008; Miller et al., 2001).

Based on a review of findings from brain research (functional/ structural neuroimaging, data from neuropsychological patients), van Elk and Aleman (2017) proposed an integrative model of the neurocognitive basis of religion and spirituality. According to this model, temporal brain areas are involved in religious visions and hallucinations, whereas multisensory integration areas (e.g., temporoparietal junction) are implicated in mystical and self‐transcendent experiences. More generally, van Elk and Aleman (2017) suggested that self‐referential processing (e.g., in the context of spiritual/religious experiences) and the belief in supernatural beings may recruit the theory‐of‐mind (ToM) network. Similarly, Kapogiannis, Barbey, Su, Krueger, et al. (2009) suggested that individuals construct representations of religious belief by differentially engaging the ToM circuit. Central hubs of this circuit include prefrontal areas, temporoparietal regions, and the hippocampus.

The current voxel‐based morphometry study focused on the belief in the miracles of Lourdes. The investigation with healthy women had a correlational approach. It was hypothesized that the degree of belief in the miracles of Lourdes would be associated with gray matter volume in the TPJ, hippocampus, amygdala, and prefrontal regions. Because many people use religious beliefs and practices to cope with stressful life events and health problems (mental, somatic), the participants were also screened for these variables. Moreover, religious–spiritual well‐being was assessed to investigate associations between the specific belief in miracles and general religiousness.

## 2 METHOD

### 2.1 Sample

A total of 84 women with a mean age of 25.4 years (*SD* = 7.6; range: 18–56) participated in the study. They had been recruited through postings of the study at the University and word of mouth. Of the participants, the majority had a high school diploma (80%). Eighty percent were University students and 11% white‐collar workers. The religious affiliation of the majority of participants was Roman‐Catholic (80%), while others stated to be Protestants (13%) or not religiously affiliated (7%). Of the participants, 45% stated that they were practicing their religion (e.g., church attendance, praying). None of the participants reported a current serious medical illness. Five of the participants reported having experienced a miracle themselves (e.g., healing of cancer, disappearance of chronic severe pain).

This study has been approved by the ethics committee of the University of Graz. Each participant provided written informed consent and received no financial compensation.

### 2.2 Questions and questionnaires

All participants answered the following questions regarding the miracles of Lourdes (“Lourdes questions”): (a) Do you think that “Lourdes water” can have positive effects?; (b) Would you use “Lourdes water” if you had a serious illness?; and (c) Do you believe in miracles (in a religious/ spiritual sense). The questions were answered on Likert scales ranging from 0 = “no” to 6 = “definitely.”

The participants also completed two questionnaires:

1. The multidimensional instrument for the measurement of religious–spiritual well‐being (MI‐RSWB 48 by Unterrainer, Huber, Ladenhauf, Wallner‐Liebmann, & Liebmann, 2010) has six subscales with each consisting of 8 items: (a) General religiousness (e.g., My faith gives me a feeling of security); (b) Forgiveness (e.g., There are things, I cannot forgive) (inverted) (c) Hope immanent (e.g., I look to the future with optimism); (d) Unity and connectedness (e.g., I have had an experience in which my person seemed to be absorbed in something bigger). (e) Hope transcendent (e.g., I often think that I must leave behind the people I love) (inverted). (f) Experience of meaning (e.g., I have the experience of the authenticity of feelings). The questions were answered on Likert scales ranging from 1 = “strongly disagree” to 6 = “strongly agree.” Cronbach's alpha of the total MI‐RSWB was 0.90 in the current sample).
2. The Brief Symptom Inventory (BSI; Franke, 2000) is a 53‐item self‐report instrument for psychological problems and their intensity during the last week (Cronbach's alpha in the current sample = 0.93). The items are rated on 5‐point scales (0 = “not at all” to 4 = “extremely”). The BSI is composed of nine symptom dimensions: somatization (e.g., chest pain and upset stomach); obsessive‐compulsive symptoms (e.g., repeated checking); interpersonal sensitivity (e.g., feelings of inferiority); depression (e.g., feeling hopeless); anxiety (e.g., nervousness); hostility (e.g., having urges to harm someone); phobic anxiety (e.g., avoidance of certain places, objects, or activities because of anxiety); paranoid Ideation (e.g., the feeling that you cannot trust others); and psychoticism (e.g., the idea that someone has power over your thoughts). In addition, a sum score (global index of distress; GSI) is computed. The BSI was included as a control variable in order to allow the exclusion of participants with tentative diagnoses of mental disorders.

The participants completed the self‐report measure during an online questionnaire screening; then, they were invited to the MRI session.

#### 2.2.1 MRI recording and analysis

The MRI session was conducted with a 3T scanner (Skyra, Siemens, Erlangen, Germany) with a 32‐channel head coil. Structural images were obtained using a T1‐weighted MPRAGE sequence (voxel size: 0.9 × 0.9 × 0.9 mm; 192 transverse slices, FoV = 224 mm, TE = 1.88 ms, TR = 1,680 ms; TI = 1,000 ms, flip‐angle = 8°). The structural scans were analyzed with the Computational Anatomy Toolbox (CAT12; v1450) implemented in SPM12 (v7487; Wellcome Trust Centre for Neuroimaging; http://www.fil.ion.ucl.ac.uk/spm/software/spm12/) in order to gain voxel‐wise comparisons of GMV.

Prior to normalization, each individual image was repositioned by setting the origin manually to the AC/PC line. Structural data were segmented into gray matter, white matter, and cerebrospinal fluid. Spatial registration was carried out by using the optimized shooting approach (Ashburner & Friston, 2011). The final resulting voxel size was 1.5 × 1.5 × 1.5 mm. Segmented gray matter images were smoothed with a Gaussian kernel with a full width at half maximum of 8 mm.

We conducted exploratory whole‐brain voxel‐based morphometry (VBM) analyses and hypothesis‐driven region of interest (ROI) analyses that focused on brain areas of the ToM network. The selected ROIs were the temporoparietal junction (TPJ), the amygdala, the hippocampus, and the medial prefrontal cortex (MPFC). We used masks with a 25% threshold derived from the Harvard–Oxford cortical structural atlas Center for Morphometric Analysis, MGH‐East). The masks were resliced to a voxel size of 1.5 × 1.5 × 1.5 mm with nearest‐neighbor interpolation.

Separate multiple regression analyses were computed to relate GMV to the “Lourdes questions” and the subdimensions of religious–spiritual well‐being. Furthermore, we computed a composite score of the three “Lourdes questions” (*M* = 1.84, *SD* = 1.5) and correlated this mean score with GMV in the selected brain regions of the ToM network. The total MI‐RSWB score (religious–spiritual well‐being) was chosen as a control variable for this analysis (partial correlation) in order to determine the specific association between belief in miracles and GMV.

Total intracranial volume (TIV), age, and an image quality index were used as covariates for each of the computed multiple regressions. To restrict the analysis to gray matter, images were thresholded for all analyses with an absolute threshold of .1. We report only results with a p‐value corrected for family‐wise‐error (FWE) below .05 (peak level; small volume correction).

## 3 RESULTS

### 3.1 Self‐report

Mean scores (standard deviations, ranges) for the “Lourdes questions” were as follows: “Belief in positive effects of Lourdes water” (*M* = 1.63; *SD* = 1.73; range = 0–6), “Use of Lourdes water for serious illness” (*M* = 2.02; *SD* = 1.62; range = 0–6), “General belief in miracles” (*M* = 1.87; *SD* = 1.79; range = 0–6). The three questions were positively correlated with each other (*r:* .57–.72).

The women obtained an average score of *M* = 191.04 (*SD* = 30.12) on the MI‐RSWB (religious–spiritual well‐being). This score was lower compared to the construction sample (*M* = 203.61, *SD* = 30.75; *t*(181.7) = −2.95; *p* = .004).

Correlations between belief in the miracles of Lourdes and (MI‐RSWB) are displayed in Table 1. Significant associations were found between the Lourdes questions (belief in positive effects of Lourdes water, use of Lourdes water for serious illness, general belief in miracles) and two subscales of the MRISB (general religiousness, feeling of unity, and connectedness). Additionally, the mean Lourdes score was positively correlated with the total MRISB score.

**Table 1:** Pearson correlations between belief in miracles of Lourdes and religious–spiritual well‐being (*n* = 84)

Lourdes questions	Belief in the positive effects of Lourdes water	Use of Lourdes water for serious illness	Belief in miracles	Mean Lourdes score*
Total score MI‐RSWB	**0.45 (<0.001)**	**0.38 (<0.001)**	**0.62 (<0.001)**	**0.56 (<0.001)**
Religiousness	**0.54 (<0.001)**	**0.46 (<0.001)**	**0.75 (<0.001)**	**0.67 (<0.001)**
Forgiveness	0.05 (0.669)	0.05 (0.630)	0.14 (0.211)	0.09 (0.405)
Hope immanent	0.07 (0.502)	0.04 (0.750)	0.14 (0.210)	0.10 (0.384)
Hope transcendent	−0.11 (0.307)	−0.02 (0.858)	0.04 (0.729)	−0.04 (0.750)
Experience of meaning	0.23 (0.039)	0.20 (0.072)	0.21 (0.061)	0.23 (0.049)
Feeling of unity and connectedness	**0.57 (<0.001)**	**0.42 (<0.001)**	**0.65 (<0.001)**	**0.63 (<0.001)**

Note

Mean: average score of the three Lourdes questions. Bold font indicates statistical significance.

The “Lourdes questions” were neither correlated with any of the BSI subscales (all *p* > .21), the total BSI (all *p* > .63) nor with age (all *p* > .16). The participants obtained BSI scores that were comparable to the healthy norm sample. Bold indicates statistically significant.

### 3.2 VBM

#### 3.2.1 Findings of the whole‐brain analysis

“Belief in the positive effects of Lourdes water” correlated positively with GMV in the postcentral gyrus. The analysis for the MI‐RSWB showed negative associations between the subscale “hope transcendent” and GMV in superior and medial temporal regions (Table 2).

**Table 2:** Association between gray matter volume and religious–spiritual well‐being (*n* = 84)

Region	*H*	*x*	*y*	*z*	*t*	*p*(FWE)	ROI/WB
Belief in miracles							
TPJ (+)	R	68	−45	10	3.69	.100	ROI
Use of Lourdes water for serious illness							
TPJ (+)	R	23	−65	54	4.31	.019	ROI
MPFC (−)	L	−2	56	12	3.95	.036	ROI
Belief in the positive effects of Lourdes water							
MPFC (−)	L/R	0	54	12	4.01	.031	ROI
Postcentral gyrus (+)	R	35	−20	56	5.21	.018	WB
Total score MRISB_R							
Hippocampus (−)	L	−33	−35	−11	3.36	.044	ROI
General religiousness							
Amygdala (−)	R	32	−6	−18	3.31	.024	ROI
Hope immanent							
Hippocampus (−)	L	−30	−33	−14	3.37	.040	ROI
Hope transcendent							
Superior temporal gyrus (−)	L	−48	−35	5	5.16	.022	WB
Posterior middle temporal gyrus (−)	L	−54	−27	5	5.08	.028	WB

Note

MNI coordinates x,y,z; p FWE (corrected for family‐wise error).

Abbreviations: (+)/(−), positive/ negative correlations; H, hemisphere (left/right); MPFC, medial prefrontal cortex; ROI/WB, results of region of interest analyses/ whole‐brain analysis; TPJ, temporoparietal junction.

#### 3.2.2 Findings of the region of interest (ROI) analyses

The three “Lourdes questions” showed the following correlations with gray matter volume (GMV) in the selected ROIs of the ToM network. “Belief in miracles” showed a marginally significant positive association with GMV in TPJ (Table 2). Within the TPJ mask, there was a significant positive correlation between GMV in the angular gyrus (MNI coordinates *x*,*y*,*z*: 68,−45,11, *t* = 3.69, *p*(FWE) = .025) and belief in miracles. “Use of Lourdes water for serious illness” correlated positively with TPJ volume and negatively with MPFC volume. “Belief in the positive effects of Lourdes water” correlated negatively with MPFC volume (Table 2).

**Figure 1:**
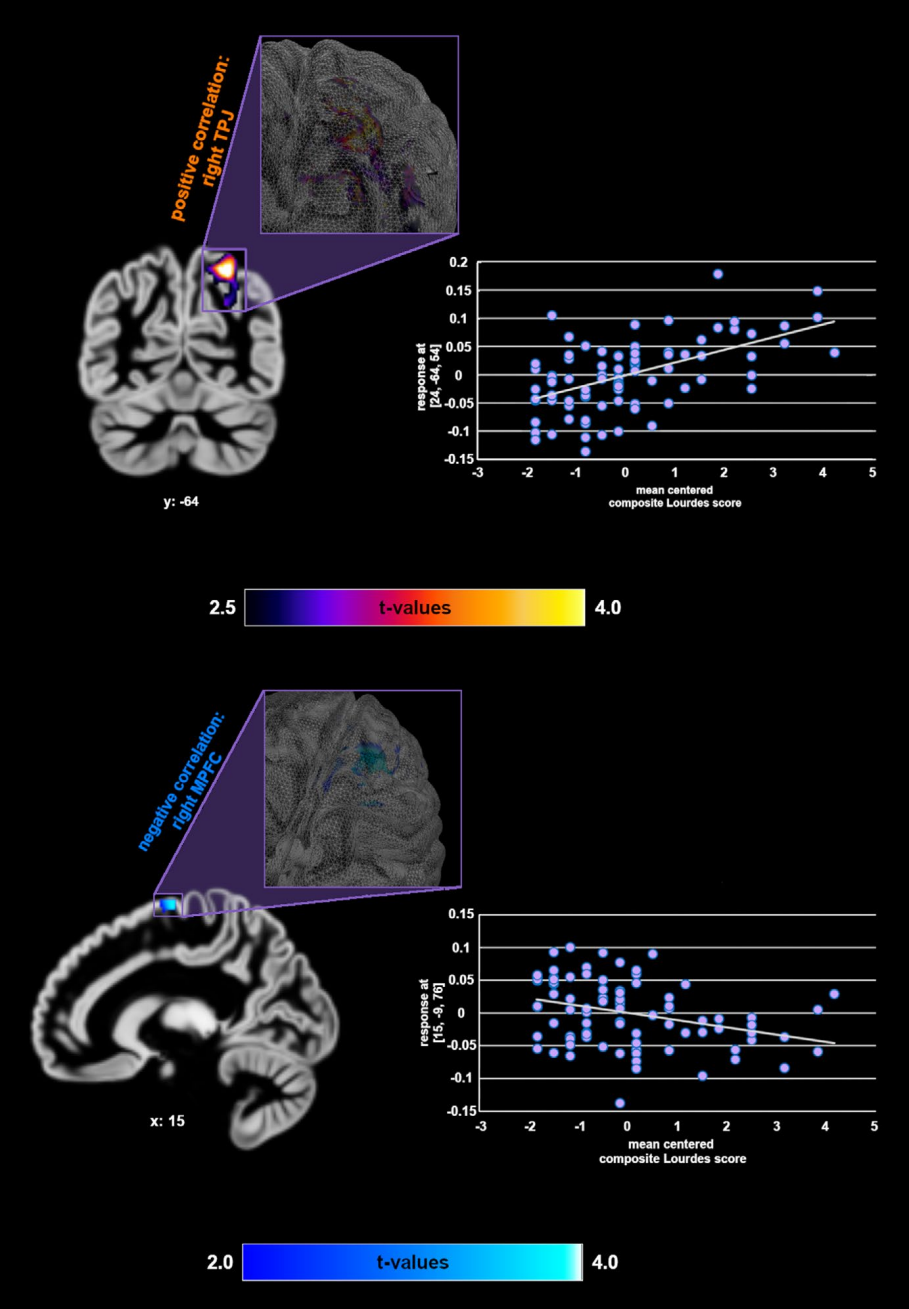
Correlations between belief in miracles (mean score of the three “Lourdes questions”) and GMV in the medial prefrontal cortex and right temporoparietal junction. Control variable = total MI‐RSWB score

Furthermore, the composite score of the three Lourdes questions (mean Lourdes score) correlated positively with GMV in the right TPJ (MNI coordinates *x*,*y*,*z*: 24,−65,54, *t* = 4.69, *p*(FWE) = .005) and negatively with GMV in the right MFPC (MNI coordinates *x*,*y*,*z*: 15,−9,77, *t* = 3.90, *p*(FWE) = .049; control variable: total MI‐RSWB (Figure 1).

The ROI analyses for the MI‐RSWB showed negative associations between hippocampus volume and the total questionnaire score as well as the subscale “hope immanent” (Table 2). General religiousness was negatively correlated with amygdala volume.

## 4 DISCUSSION

The present VBM study revealed that belief in the miracles of Lourdes correlated positively with gray matter volume (GMV) in the temporoparietal junction (TPJ) and negatively with GMV in the medial prefrontal cortex (MPFC).

The TPJ is roughly characterized as an area at the border between the temporal and parietal lobes surrounding the ends of the Sylvian fissure. The TPJ receives information from the thalamus and limbic regions, as well as from sensory systems (for a review see Donaldson, Rinehart, & Enticott, 2015.) The TPJ integrates information from both the external environment and from within the body. This region is activated when we ascribe mental states (e.g., beliefs) to other people but also during self‐referential processing (e.g., mentalizing about our own beliefs; see meta‐analysis by Schurz, Radua, Aichhorn, Richlan, & Perner, 2014). The mentioned functions are all connected to theory of mind (ToM). ToM refers to our ability to understand mental states such as intentions, goals, and beliefs of others and ourselves (Singer, 2008).

It has been shown that noninvasive stimulation of the TPJ is able to alter self‐awareness (e.g., in the form of so‐called “out‐of‐body experiences” or perceptions of an “illusory shadow person”). TPJ stimulation can also change belief reasoning, moral judgments, and other tasks related to social cognition (Donaldson et al., 2015). Social cognition involves attentional processes and multisensory testing of internal predictions (Decety & Lamm, 2007). This function is important in the context of belief in miracles.

The ToM network also includes the medial prefrontal cortex (MPFC; see Schurz et al., 2014). Activation of this region has been consistently recognized in early studies on ToM, which led to the initial assumption that the MPFC is specifically linked to reasoning about belief (e.g., Gallagher & Frith, 2003, Leslie et al., 2004). However, more recent views describe the MPFC to be more generally involved in the processing of socially and emotionally relevant information about others and the self (e.g., Saxe & Powell, 2006). Moreover, the MPFC has been conceptualized as a core brain area involved in self‐control (Tang et al., 2015). A stronger belief in miracles by the power of God might be associated with a reduced sense of self‐control. In line with this assumption are findings by Newton and McIntosh (2009) who revealed that the perception that God was in control was related negatively to the perception of self‐control. Therefore, a negative correlation between MPFC volume and belief in miracles seems plausible.

In conclusion, we found two regions of the neural ToM network to be associated with beliefs in the miracles of Lourdes. This observation fits nicely to claims that religious experiences and beliefs are associated with general cognitive processes related to self‐regulation, attention control, and self‐awareness (Azari et al., 2001; Schjoedt, 2009; Tang et al., 2015). There are no specific religious networks in the brain but religious experience is subserved by common neural functions. Religious attitudes involve attribution of meaning. The meaning of a miracle is that one can rely on a powerful, beneficent, supernatural being (e.g., God) that can intervene and help to cope with extremely difficult situations (e.g., involving serious medical illness). This interpretation is in line with the self‐reports of the participants. The belief in the miracles of Lourdes was positively correlated with general religiousness (faith that gives a feeling of security) as well as with the feeling of unity and connectedness.

The current VBM study identified two additional whole‐brain findings; the belief in the positive effects of Lourdes water was positively correlated with gray matter in the postcentral gyrus, the location of the somatosensory cortex. Moreover, one subscale of the MI‐RSWB “hope transcendent” correlated negatively with GMV in the superior and medial temporal gyrus, which are involved in social cognition processes and religious visions (van Elk & Aleman, 2017). Other correlations identified referred to negative associations between hippocampus volume and the total MI‐RSWB as well as the subscale “hope immanent.” These correlations were not in the predicted direction. However, the negative correlation between amygdala volume and general religiousness is in line with previous research (e.g., Tang et al., 2015) demonstrating that the practice of religiously inspired techniques is associated with lower amygdala volume.

We also need to mention the following limitations of the current investigation. We only investigated women; therefore, our results cannot be generalized to men. Further, we studied a rather skeptic group with regard to belief in miracles. Previous surveys indicated that spiritual and religious beliefs of many people include the existence of miracles. For example, in a large survey in the United States (*n* = 36,000) 78% of people under the age of 30 stated to believe in miracles (Pew Research Center, 2010). Over 80% of those with Protestant and Catholic affiliations endorsed this belief. In contrast, in our sample, the belief in miracles was below average; on a scale ranging from 0 to 6 (6 = definite belief in miracles), the average rating was 1.87. For future investigations, it would, therefore, be promising to investigate two extreme groups with a high versus low degree of belief in miracles.

## CONFLICT OF INTEREST

None declared.

## Data Availability

The data that support the findings of this study are available from the corresponding author upon reasonable request.
